# Differences in intimate partner violence screening preconception to postpartum for US‐born versus foreign‐born birthing people

**DOI:** 10.1002/pmf2.70311

**Published:** 2026-05-14

**Authors:** Elizabeth Mirsky, Ashley Boerrigter, Gregory Hawk, Emily DeFranco

**Affiliations:** ^1^ Department of Obstetrics and Gynecology Division of Maternal Fetal Medicine University of Kentucky College of Medicine Lexington Kentucky USA; ^2^ Dr. Bing Zhang Department of Statistics University of Kentucky College of Arts and Sciences Lexington Kentucky USA

**Keywords:** foreign‐born birthing people, intimate partner violence, IPV screening, postpartum, PRAMS, pregnancy

## Abstract

**Introduction:**

This study aimed to investigate whether socioeconomic barriers to healthcare unique to foreign‐born birthing people in the United States are reflected in reduced frequency of screening for intimate partner violence (IPV) in the preconception, pregnancy, and postpartum periods.

**Methods:**

This was a cross‐sectional study using Pregnancy Risk Assessment Monitoring System (PRAMS) data from 2020 to 2021. PRAMS collected data on screening for physical and emotional IPV before, during, and after pregnancy, as well as the experience of IPV before and during pregnancy. The primary outcomes, frequency of reported IPV experience and having been screened for IPV, were compared between foreign‐born versus US‐born status and relative risks (RRs) were calculated. Weighted analyses of PRAMS data were employed to be reflective of representative state populations using predefined methods.

**Results:**

A total of 63,761 participants were included. Sociodemographic characteristics differed between US‐born and foreign‐born status. Approximately one in four individuals reported not being screened for IPV during pregnancy, with screening rates lower before and after pregnancy. Among those who reported experiencing IPV, over one‐third were not screened. Foreign‐born respondents were overall less likely to be screened for IPV before pregnancy than US‐born respondents, 49.3% versus 54.3% (RR, 0.84 [0.78–0.91]), but more likely to be screened during and after pregnancy. There was also no significant association between being screened for IPV and foreign‐born status among those reporting having previously experienced IPV before and during pregnancy (RR, 0.80 [0.47–1.34] and RR, 1.60 [0.90–2.84], respectively).

**Conclusion:**

Screening for IPV during pregnancy remains inadequate, with approximately one in four individuals reporting not being screened. While multiple barriers to IPV screening exist, foreign‐born status was not associated with lower rates of IPV screening during or after pregnancy. This finding should be interpreted with caution, considering various barriers related to birthing status that cannot be adequately evaluated using PRAMS. This also highlights the importance of IPV screening among all pregnancies, regardless of perceived risk status.

## 1 INTRODUCTION

Intimate partner violence (IPV) during pregnancy has been associated with adverse maternal and neonatal outcomes, including increased risk of preterm birth and low birth weight, among others. Physical violence during pregnancy is the form of IPV most likely to be associated with adverse outcomes [1]. Several organizations, such as the United States Preventive Services Task Force (USPSTF) [2] and the American College of Obstetricians and Gynecologists (ACOG) [3], promote universal screening for IPV for all pregnant and postpartum individuals. Multiple factors are associated with the likelihood of completing perinatal IPV screening, including maternal age, education, and income status. Unfortunately, the prevalence of screening remains low nationwide [4]. In addition, being part of a minority group has been shown to be a risk factor for IPV in nonpregnant persons [5]. There is a paucity of research on the effect of foreign‐born status on IPV screening in pregnancy. This study aimed to investigate whether socioeconomic barriers to healthcare unique to foreign‐born birthing people in the United States are reflected in reduced frequency of screening for IPV in the preconception, pregnancy, and postpartum periods.

## 2 MATERIALS AND METHODS

This was a cross‐sectional study using Pregnancy Risk Assessment Monitoring System (PRAMS) data from 2020 to 2021 from 40 and 35 US states, respectively [6]. The PRAMS questionnaire was developed by the US Centers for Disease Control and Prevention (CDC) and consists of questions about preconception and prenatal care, insurance status, tobacco and substance use, physical abuse, infant health care and breastfeeding, and contraceptive use. For this study, questions on screening for physical and emotional IPV before, during, and after pregnancy, as well as experience of IPV before and during pregnancy, were included. Physical and emotional IPV were combined to create the defined outcome variables. Participants were asked whether they were screened for IPV at different points prior to, during, and after pregnancy, as well as whether they had experienced IPV. The primary outcomes were the frequency of reported IPV experience and the report of undergoing screening for IPV. The exposure variable was birthplace (foreign‐born vs. US‐born status), although duration of residence in the United States, language preference, and country of origin could not be captured using the questionnaire. Demographic characteristics included maternal age, race, level of education, insurance status, parity, obesity (defined as body mass index [BMI] ≥ 30), tobacco use, and the presence of pregnancy comorbidities including chronic hypertension and diabetes.

Differences in categorical outcomes between groups were compared using chi‐square tests, while the unadjusted relative risk (RR) for these outcome measures was estimated using generalized linear models (GLMs) with a binomial error distribution and a log link function. RR was reported with 95% confidence intervals (CIs). Weighted analyses of PRAMS data were employed to be reflective of representative state populations using predefined methods [7]. This allows for the generalization of results to an entire population of births and employs complex sampling and statistical designs to account for sampling, nonresponse, and noncoverage rates.

This study followed the Strengthening the Reporting of Observational Studies in Epidemiology (STROBE) guidelines and was exempt from oversight by the Institutional Review Board (IRB) at the institution, as it was based on publicly available, deidentified data and did not meet criteria for human subjects research.

## 3 RESULTS

There were 63,761 participants in the study period from participating US states. All were people who had given birth in the last 2 to 6 months to a live‐born infant. Sociodemographic characteristics, including maternal age, race, level of education, and insurance status, significantly differed between US‐born and foreign‐born status (Table 1). US‐born participants were on average younger, more likely to be of white race, had higher degrees of education, and were more likely to have private insurance than foreign‐born participants. US‐born participants were also more likely to receive the recommended number of prenatal care visits. Results are reported as unadjusted estimates. Approximately three out of four participants reported being screened for IPV during pregnancy, with lower IPV screening rates before and after pregnancy among all participants (53.6% and 61.8%, respectively). Foreign‐born participants were overall less likely to be screened for IPV before pregnancy than US‐born participants (49.9% and 54.3%; RR, 0.84 [0.78–0.91]), but more likely to be screened during and after pregnancy (Table 2). US‐born participants were more likely to experience IPV before and during pregnancy than foreign‐born participants (1.5% vs. 0.9%; RR, 0.62 [0.47–0.80] and 1.2% vs. 0.9%; RR, 1.09 [1.02–1.18]); however, there was no significant association between being screened for IPV and foreign‐born status among those reporting having experienced IPV before or during pregnancy.

**TABLE 1:** Sociodemographic and clinical characteristics by birthplace.

	Birthplace		
Characteristic	United States	Foreign country	*p* value
Study total, number (%)	2,469,865 (81)	569,594 (19)	
	51,646^a^	12,115^a^	
Maternal Age			
<18	1.2	0.7	
18–34	74.4	64.2	
>34	17.5	27.8	
Race			
Asian	1.4	22.4	
Black	14.0	11.6	
Hispanic	11.8	45.0	
White	68.7	15.9	
None of the above	4.1	5.2	
Level of education			
<High school (HS) diploma	8.2	23.4	
HS diploma/GED	24.7	25.1	
Some college	27.5	18.3	
Bachelor's degree	24.0	18.9	
Graduate degree	15.5	15.4	
Use of WIC	28.7	39.2	
Insurance status			
Private insurance	57.1	39.0	
Medicaid	37.4	52.8	
Self‐pay	2.2	4.3	
Other	1.0	1.4	
Primiparous	32.2	32.2	0.949
Obesity, BMI ≥ 30 (prepregnancy)	33.6	24.9	
Tobacco use	7.4	0.6	
Chronic hypertension	3.1	2.0	
Prepregnancy diabetes	1.1	1.3	0.262
Gestational diabetes	7.1	12.7	
Infertility treatment	2.3	2.2	0.353
Prenatal care			
Limited (≤5 visits)	7.1	9.8	
Moderate (6–8 visits)	22.4	27.4	
Recommended ≥9	70.5	62.8	

*Note*: Data in the cells of Table 1 are percentages of the *n* reported at the top of the table and reflect weighted analyses. All *p* values are <0.0001 and, therefore, statistically significant, unless otherwise stated.

Abbreviations: BMI, body mass index; GED, General Educational Development; WIC, women, infants, and children.

^a^ Unweighted study total

**TABLE 2:** Prevalence of intimate partner violence and screening by birthplace.

Birthplace				
	All	United States	Foreign country	RR (95% CI)
Prepregnancy	2,115,360 44,375^a^	1,798,386 37,726^a^	316,974 6649^a^	
Screened for IPV, overall population	53.6	54.3	49.9	0.84 (0.78–0.91)
Reported IPV % of reported screened	1.4 61.4	1.5 68.4	0.9 63.2	0.62 (0.47–0.80) 0.80 (0.47–1.34)
Pregnancy	2,962,280 62,141^a^	2,419,923 50,764^a^	542,357 11,377^a^	
Screened for IPV, overall population	74.6	74.3	76.0	1.09 (1.02–1.18)
Reported IPV % of reported screened	1.1 73.1	1.2 74.7	0.9 87.4	0.81 (0.61–1.07) 1.60 (0.90–2.84)
Postpartum	2,672,841 56,069^a^	2,195,787 46,062^a^	477,054 10,007^a^	
Screened for IPV, overall population	61.8	59.5	72.3	1.78 (1.65–1.91)

*Note*: Data in the cells of Table 2 are percentages of the *n* reported in the top row of the section.

Abbreviations: CI, confidence interval; IPV, intimate partner violence; RR, relative risk.

^a^ Unweighted study total.

## 4 DISCUSSION

Approximately one in four individuals reported not being screened for IPV during pregnancy. Rates of screening were even lower before and after pregnancy. Among those who reported experiencing IPV, over one‐third were not screened. While multiple barriers to IPV screening exist, foreign‐born status was not associated with lower rates of IPV screening during or after pregnancy. This finding should be interpreted carefully, taking into consideration that it is unlikely that there is a true absence of barriers, but rather that additional factors may not be accounted for in the assessment of IPV screening in these differing populations. Healthcare utilization patterns, pregnancy‐related insurance coverage, and differing institutional screening mandates may impact the ability to accurately analyze rates of screening. This highlights the importance of IPV screening among all pregnancies, regardless of perceived risk status.

These rates of IPV screening during pregnancy corroborate with prior studies, which have found rates as low as ∼50% in a similar PRAMS population with various important predictors for screening including race, education, and insurance status [8]. Another study explored rates of IPV and screening among rural and urban birthing people and found that rural IPV victims are at higher risk of not being screened [9]. The results of our study, when combined with various prior studies, reveal that there is significant room for improvement in universal IPV screening during prenatal and postpartum healthcare.

A strength of this study is the examination of foreign‐born birthing status as the primary independent variable in the setting of screening for IPV. A previous study using PRAMS reported on the prevalence of pregnancy‐related hypertensive disorders among foreign‐born versus US‐born birthing people [10]. Similarly, another study reviewed the effects of ethnicity, nativity, and country of foreign birth on preterm birth rates in the setting of stressful life events [11]. These studies bring to light the potential importance of birthing status in association with multiple predictors of maternal and neonatal outcomes, among which IPV is an important consideration.

Limitations of the study include the small effect size in the setting of a large sample size, which contributes to statistical significance but questionable clinical significance. Additionally, the PRAMS questionnaire is subject to recall bias when obtaining information regarding sensitive topics, especially when administered in survey form rather than by telephone. The route for obtaining questionnaire answers is not reported and therefore unable to be considered in statistical modeling. In addition, only live births are included, which may exclude pregnancies with adverse outcomes as a result of severe IPV resulting in selection bias. This systematic exclusion may lead to underestimating the association between IPV and adverse outcomes and limit the generalizability to participants with the highest risk profiles. Finally, language barriers and cultural norms may be important confounders in this study and were difficult to adjust for with the available data. The primary exposure variable, US‐born versus foreign‐born status, is broad and important factors such as duration of residence in the United States, language preference, and country of origin cannot be evaluated or adjusted for using PRAMS.

## 5 CONCLUSION

Screening for IPV during pregnancy remains inadequate, with approximately one in four individuals reporting not being screened. Rates of screening may be even lower in those who have previously experienced IPV. While foreign‐born status does not appear to be associated with IPV screening rates, overall screening remains inadequate. Future studies should further review the modifiable risks for inadequate screening in an attempt to improve universal IPV screening during the preconception, prenatal, and postpartum periods.

## FUNDING INFORMATION

The authors received no specific funding for this work.

## CONFLICT OF INTEREST STATEMENT

The authors declare no conflicts of interest.
